# Supplementary material for: Celda: a Bayesian model to perform co-clustering of genes into modules and cells into subpopulations using single-cell RNA-seq data
Source: NAR Genom Bioinform. 2022 Sep 13;4(3):lqac066. doi: 10.1093/nargab/lqac066 (PMC9469931; doi:10.1093/nargab/lqac066)
Supplement: lqac066_Supplemental_Files [file lqac066_supplemental_files.zip › 20220701_celda_Supplement.pdf]

Table S1. Marker genes used to identify cell types in the PBMC dataset.

Cell type	Celda_CG cell cluster	Marker genes
Plasma cell	1	IGHG1, IGHG3, IGLC2, IGLC3
B-cell	2, 3, 4	CD19, MS4A1, CD79A, CD79B
Dendritic cell	5, 6	FCER1A, CLEC10A, FLT3, HLA-DPB1, HLA-DPA1, HLA-DQA1
Plasmacytoid dendritic cell	7	ITM2C, IRF7, IRF8, LILRA4, CLEC4C
CD34+ progenitor cell	8	CD34, SOX4, MYB, GATA2
Natural killer cell	9	NKG7, KLRD1, CST7
Megakaryocyte	10	PPBP, ITGA2B, PF4
CD14+ monocyte	11, 12, 13	CD14, S100A9, S100A8
FCGR3A+ monocyte	14	FCGR3A, LST1, SERPINA1
Proliferating T-cell	15	MKI67, IL2RA, CENPF, CENPM
Memory T-cell	16	CCR7, SELL, CD27
Naive $CD8^+$ T-cell	17	CCR7, CD3D, CD8A, CD8B
Natural killer T-cell	18	CD3D, GNLY, KLRG1, GZMA, GZMH
Cytotoxic T-cell	19	CD3D, CD8A, CD8B
T helper cell	20	CD3D, CD4, IL7R

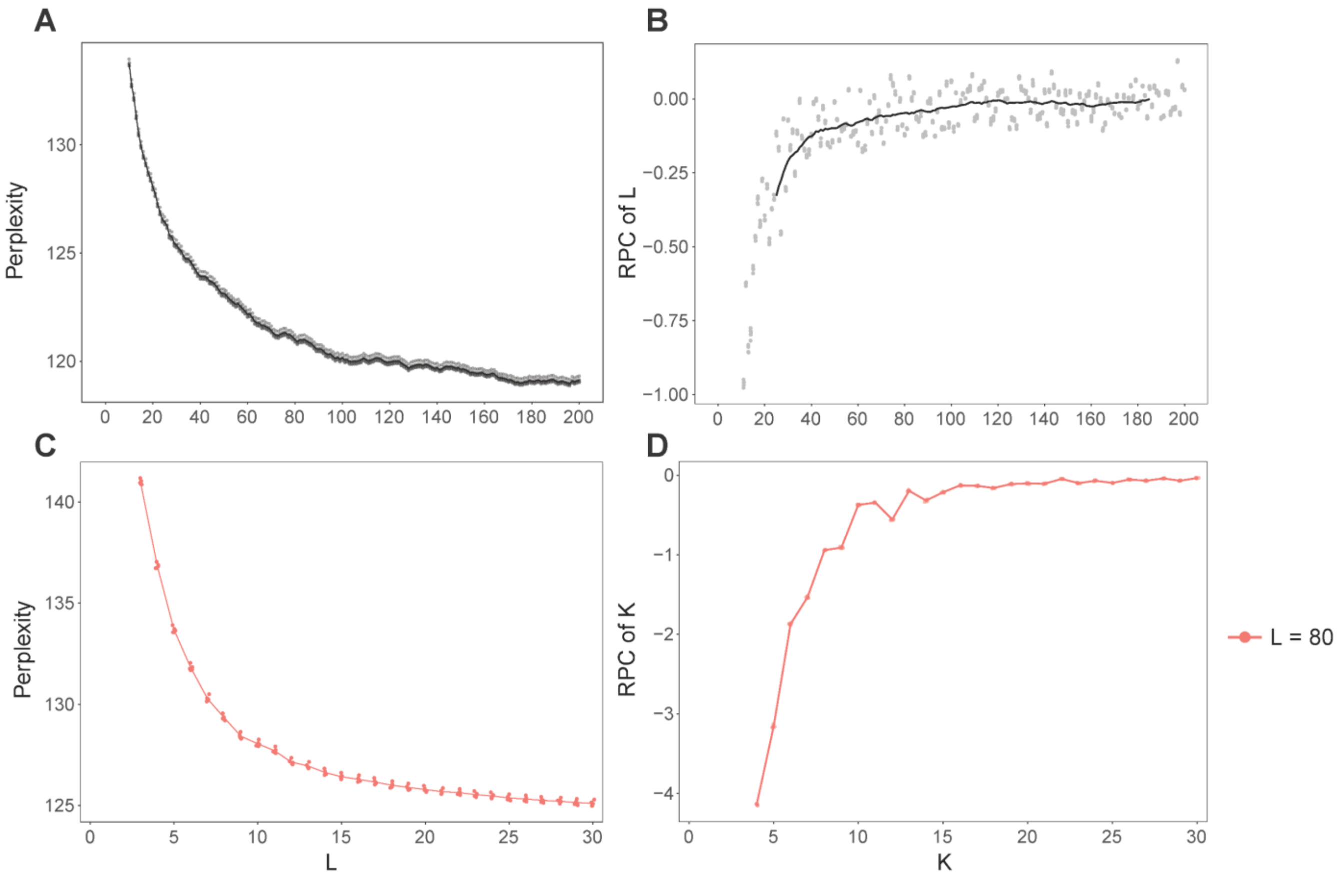

Figure S1. Determining the optimal number of transcriptional modules (L) and cell clusters (K) for the PBMC 4K dataset. a, Scatter plot showing the perplexity of models with a range of 10 to 200 transcriptional modules. **b**, Scatter plot showing the rate of perplexity change (RPC) between each model with L transcriptional modules and the previous model with $L - 1$ transcriptional modules. The solid black line represents moving average of centered rolling windows of size 30. For the PBMC 4K dataset, a L value of 80 was selected because it was beyond the "elbow" on the curve and captured biologically relevant modules observed when performing manual review of module heatmaps. **c**, Scatter plot showing the perplexity of models with a range of 3 to 30 cell clusters with L fixed at 80. **d**, Scatter plot showing the RPC between each model with K cell clusters and the previous model with $K - 1$ cell clusters. For the PBMC 4K dataset, a K value of 20 was selected because it was beyond the "elbow" on the curve and captured known and novel cell types.

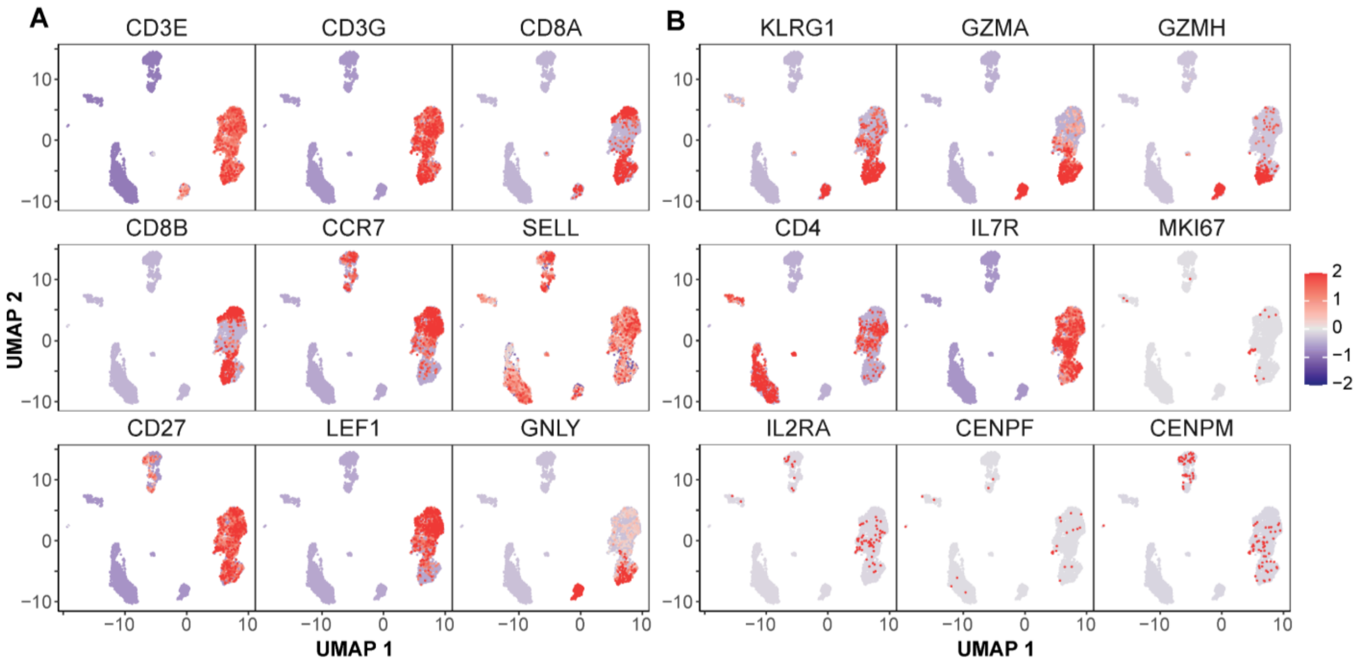

Figure S2. UMAPs showing expression of marker genes for T cell subpopulations. a, UMAPs colored by scaled normalized expressions of T cell markers CD3E, CD3G, cytotoxic T cell markers CD8A, CD8B, naive T cell markers CCR7, SELL, CD27, LEF1, and NK T cell marker GNLY. **b**, UMAPs colored by scaled normalized expressions of NK T cell markers KLRG1, GZMA, GZMH, T helper cell markers CD4, IL7R, and activated T cell markers MKI67, IL2RA, CENPF, CENPM.

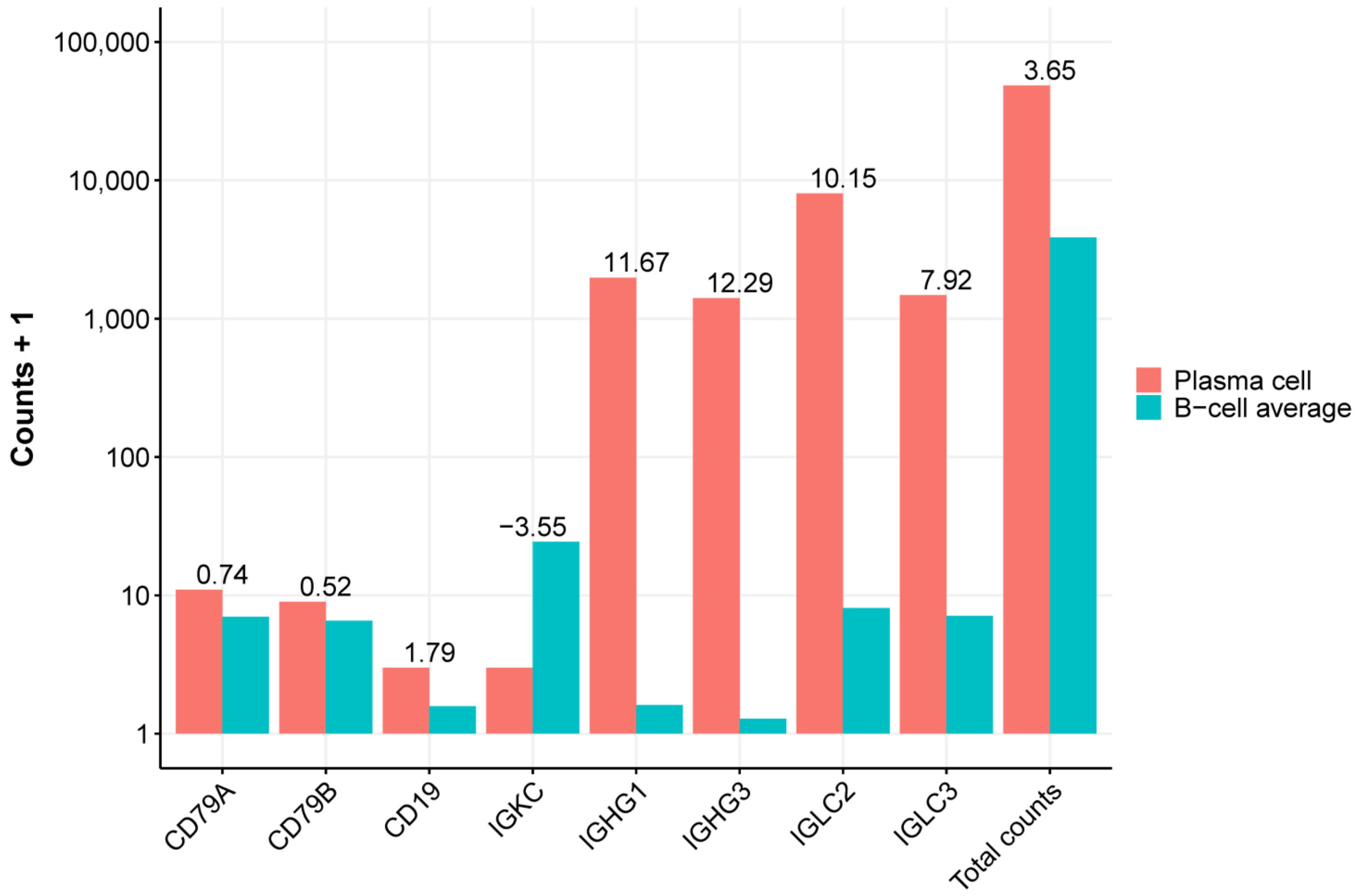

Figure S3. The plasma cell showed much higher expression of IGHG1, IGHG3, IGLC2, and IGLC3 compared to B-cells. The total raw UMI counts (with one pseudocount) of CD79A, CD79B, CD19, IGKC, IGHG1, IGHG3, IGLC2, and IGLC3 for the plasma cell (cell cluster 1) and B-cells (cell clusters 2, 3, 4) are shown on the bar plots. The $\log_2$ fold change values are shown on the top of bars. The plasma cell showed much higher expression levels of IGHG1, IGHG3, IGLC2, and IGLC3 while having relatively similar expression levels of CD79A, CD79B, and CD19 compared to the average of B-cells.

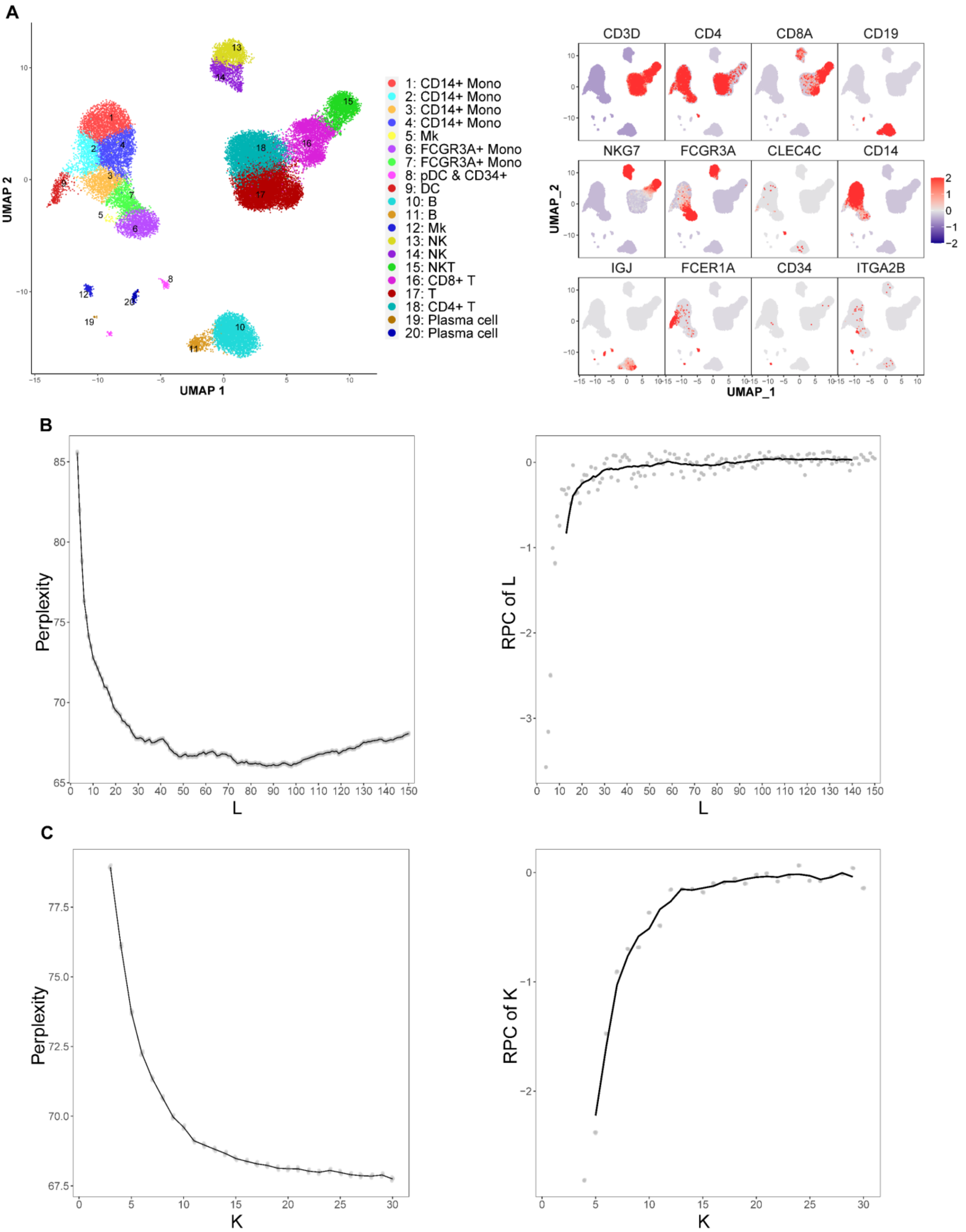

Figure S4. Celda identifies immune cell subpopulations in PBMC 33k dataset. a, UMAP of PBMCs colored by cell populations identified by Celda (left) and by expressions of selected marker genes (right). Scaled normalized expressions of representative gene markers show clustering of cell subpopulations including T-cells (CD3D), $CD4^{+}$ T-cells (CD4), $CD8^{+}$ T-cells (CD8A), B cells (CD19), natural killer cells (NKG7), FCGR3A+ monocytes (FCGR3A), plasmacytoid dendritic cells (CLEC4C), CD14+ monocytes (CD14), plasma cells (IGJ), dendritic cells (FCER1A), CD34+ progenitor cells (CD34), and megakaryocytes (ITGA2B). **b**, Scatter plot showing the perplexity and RPC of models with a range of 3 to 150 transcriptional module (L). A L value of 80 was selected. **c**, Scatter plot showing the perplexity and RPC of models with a range of 3 to 30 cell clusters (K) with L fixed at 80. A K value of 20 was selected.

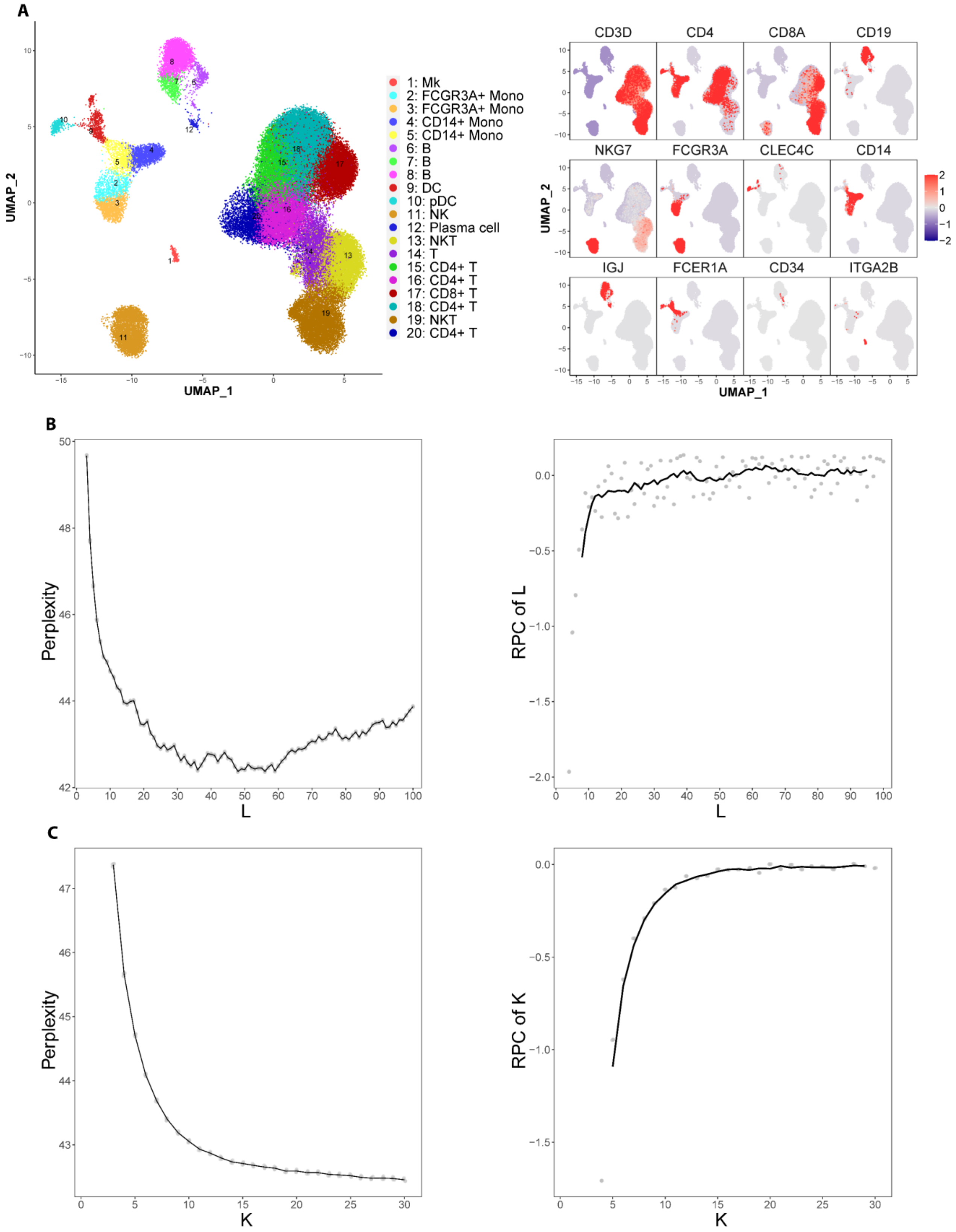

Figure S5. Celda identifies immune cell subpopulations in PBMC 68k dataset. a, UMAP of PBMCs colored by cell populations identified by Celda (left) and by expressions of selected marker genes (right). Scaled normalized expressions of representative gene markers show clustering of cell subpopulations including T-cells (CD3D), $CD4^{+}$ T-cells (CD4), $CD8^{+}$ T-cells (CD8A), B cells (CD19), natural killer cells (NKG7), FCGR3A+ monocytes (FCGR3A), plasmacytoid dendritic cells (CLEC4C), CD14+ monocytes (CD14), plasma cells (IGJ), dendritic cells (FCER1A), CD34+ progenitor cells (CD34), and megakaryocytes (ITGA2B). **b**, Scatter plot showing the perplexity and RPC of models with a range of 3 to 100 transcriptional module (L). A L value of 80 was selected. **c**, Scatter plot showing the perplexity and RPC of models with a range of 3 to 30 cell clusters (K) with L fixed at 80. A K value of 20 was selected.

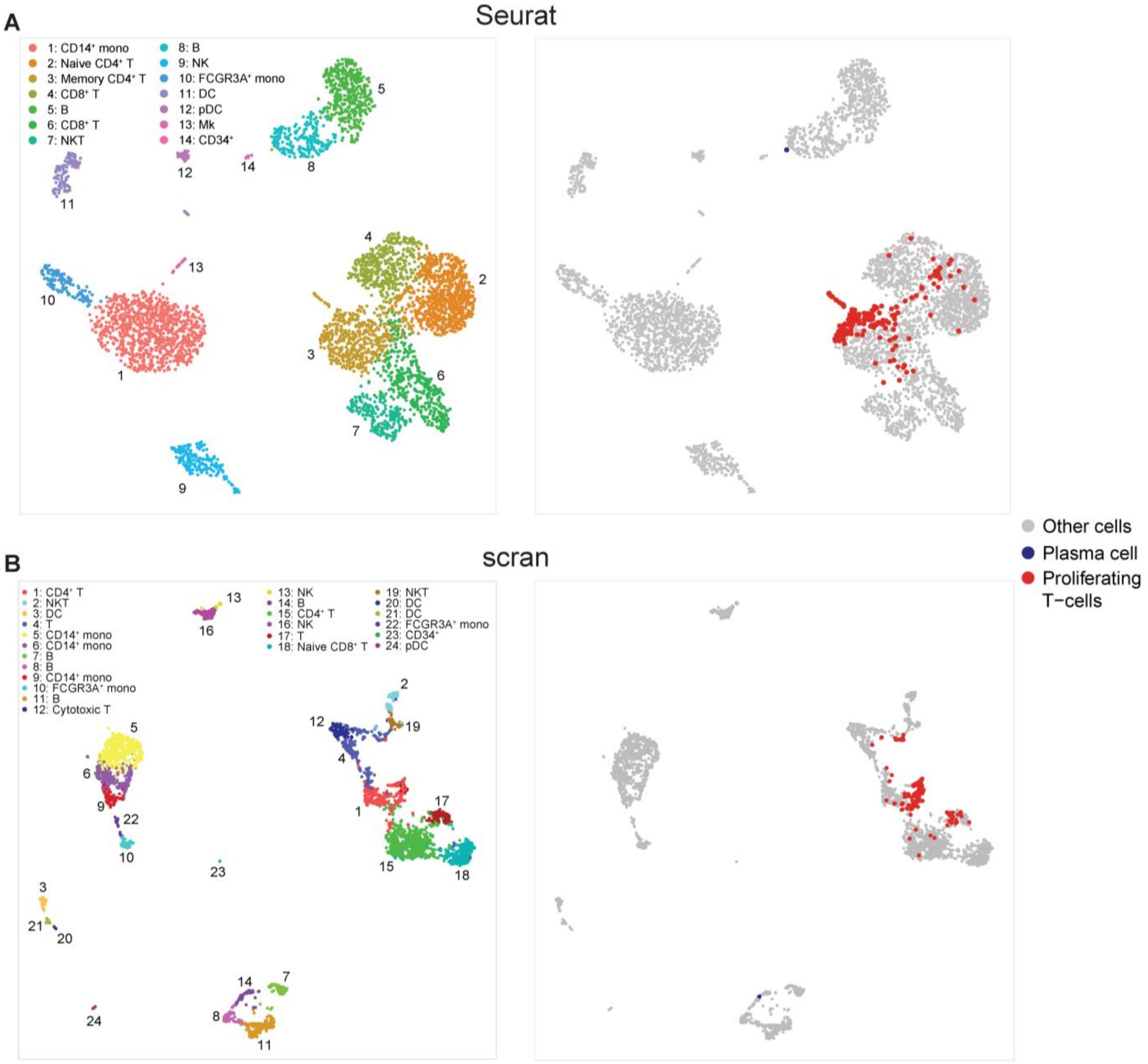

Figure S6. The Proliferating T-cells and plasma cell are not distinctly clustered by Seurat and scran. UMAPs of PBMCs colored by cell populations identified by Seurat and scran are shown. The proliferating T-cells and plasma cell identified by Celda are highlighted on the right. **a**, Seurat grouped both the proliferating T-cells and the plasma cell in the $CD4^+$ T-cell cluster. **b**, scran grouped the proliferating T-cells in the $CD4^+$ T-cell cluster and clustered the plasma cell with B-cells.

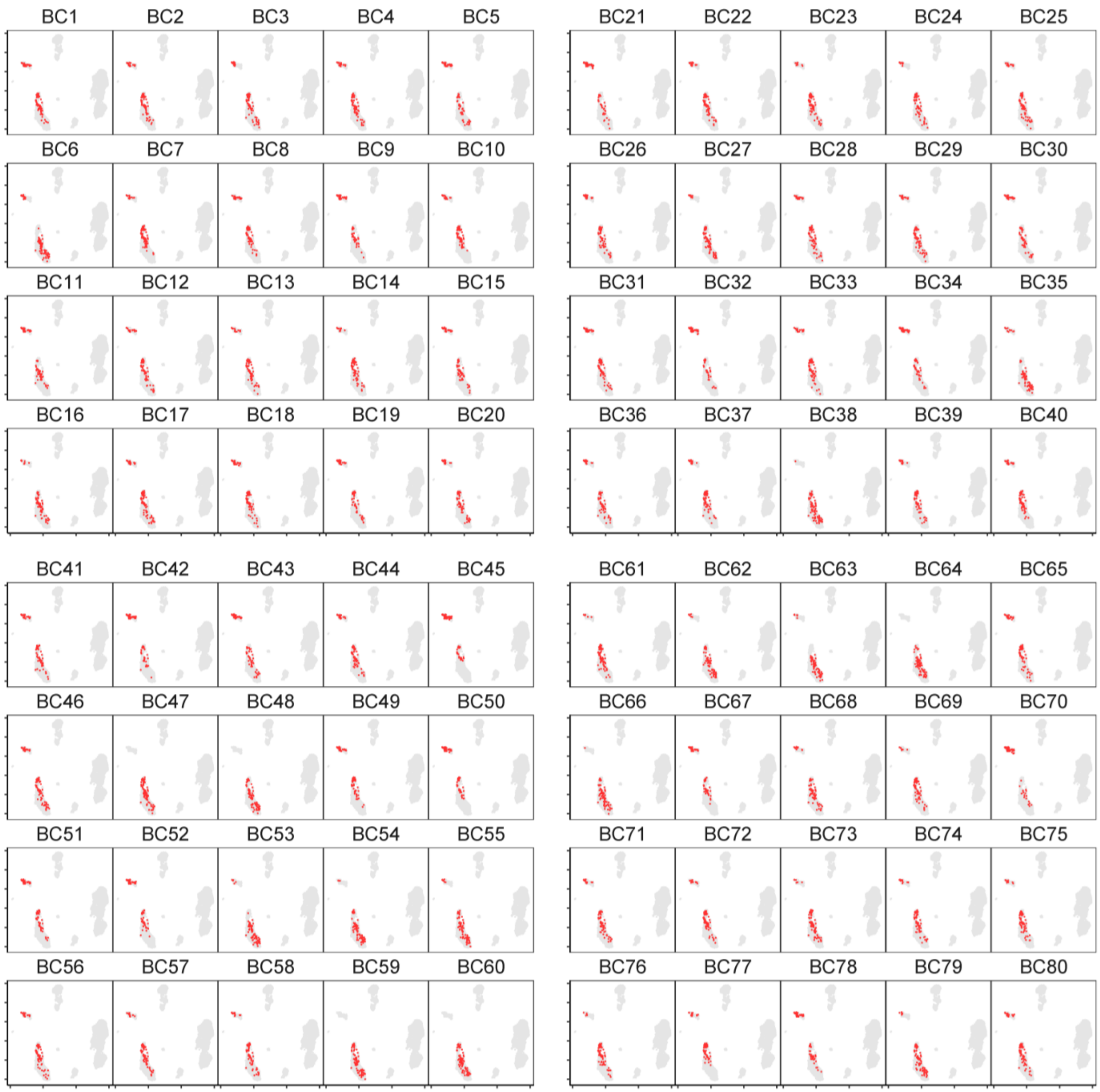

Figure S7. QUBIC2 only identified dendritic and monocyte cell subpopulations. UMAPs of PBMCs colored by cell populations identified by the 80 QUBIC2 biclusters are shown. All of the cells in each of the 80 biclusters identified by QUBIC2 were from the dendritic and/or monocyte subpopulations and no biclusters contained B cells, T cells or natural killer cells, pDCs, or the novel subpopulations of proliferating T-cells.

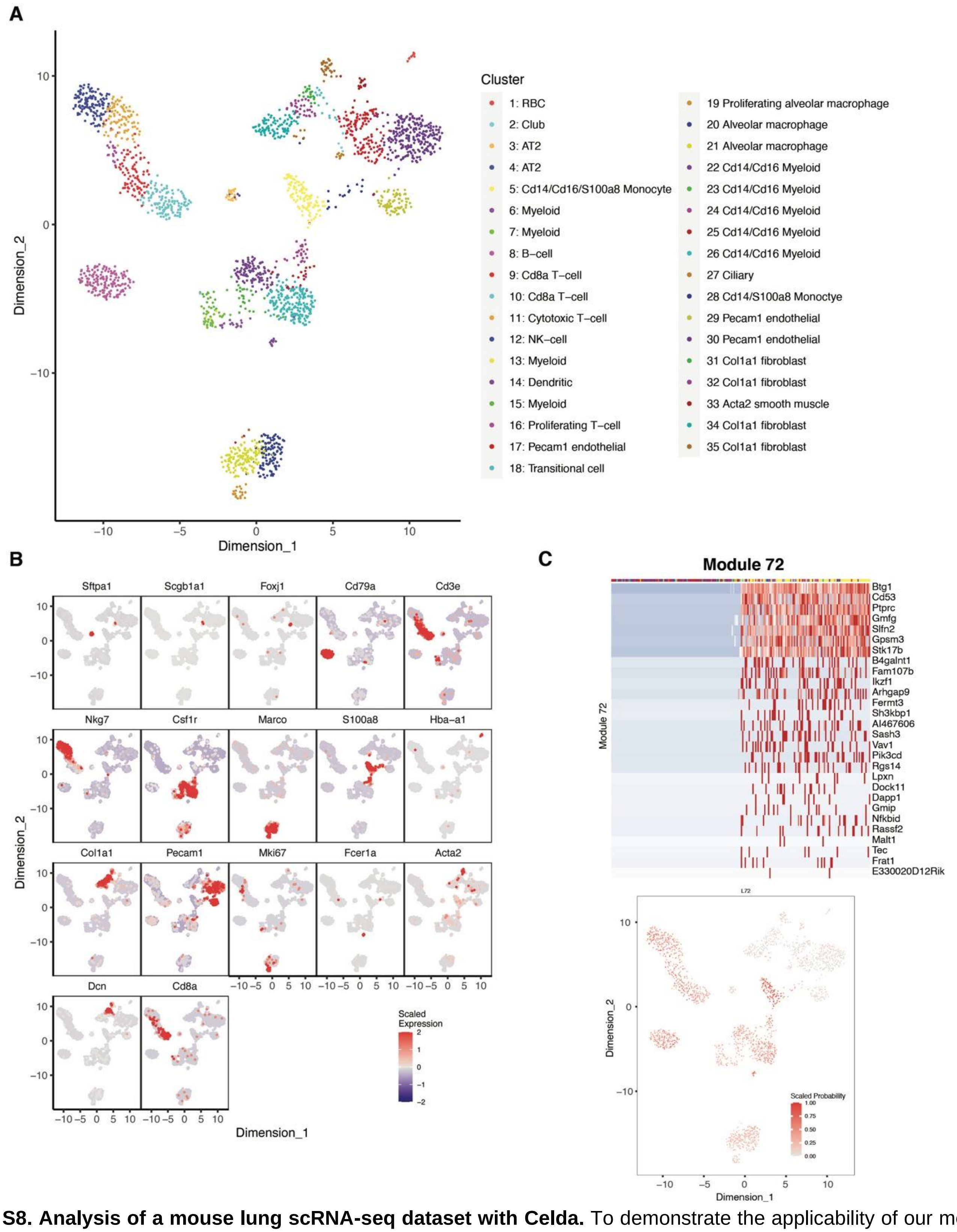

Figure S8. Analysis of a mouse lung scRNA-seq dataset with Celda. To demonstrate the applicability of our methods to additional data sets, we applied celda to a mouse lung dataset generated from the Tabula Muris consortium (n = 2,150 cells). We identified 125 modules and 35 cell populations using the recursive splitting procedures. **A,** Cells are plotted on a UMAP generated using celda module probabilities and colored by cluster label. **B,** The major cell type of each cluster was identified by examining expressing of known marker genes (AT2: Sftpa1, Club: Scgb1a1, Ciliary: Foxj1, B-cell: Cd79a, T-cell: Cd3e and Cd8a, NK-cell: Nkg7, Myeloid: Csf1r, Alveolar macrophage: Marco, Monocyte: S100a8, Red blood cell: Hba-a1, Fibroblast: Col1a1, Endothelial cells: Pecam1, Proliferating cells: Mki67, Dendritic cells: Fcer1a, Smooth muscle cells: Acta2 and Dcn. **c,** Many modules were identified that uniquely identify a cell cluster or major cell type. A module contains Ptprc (also known as Cd45), a known marker for immune cells, is shown as an example of a module that spans across cell types.

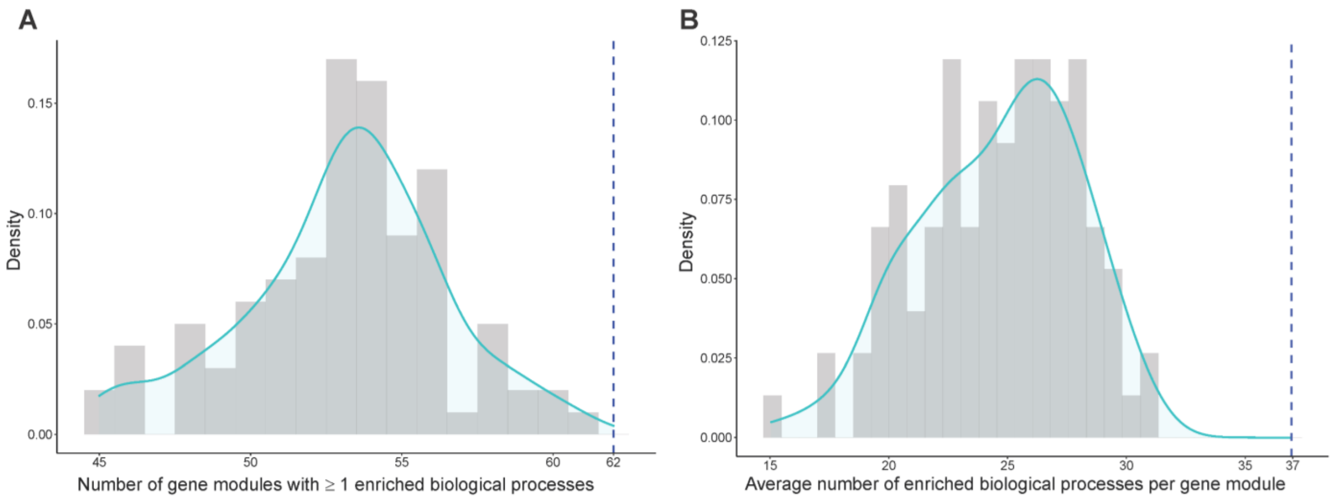

Figure S9. Genes in Celda gene modules are biologically relevant. To demonstrate that the gene modules identified by Celda_CG are biologically meaningful, we tested the enrichment of the genes from each of the 80 lists of gene modules in "GO_Biological_Process_2021" database using Enrichr. 100 random permutations of gene module labels were performed to generate null distributions. **A,** The histogram and density plot show, out of total 80 gene modules, the number of gene modules with at least one enriched GO terms from the 100 random permutations. The blue vertical dashed line represents the number from true gene modules identified by Celda_CG. 62 of the 80 modules were enriched for at least one biological process pathway. **B,** The histogram and density plot show the average number of enriched biological processes per gene module from the 100 random permutations. The blue vertical dashed line represents the number from true gene modules identified by Celda_CG. The 80 modules have 37.0 enriched GO terms on average. Overall, these results demonstrate that genes within each module identified by Celda_CG are more enriched for biological terms than expected by chance.

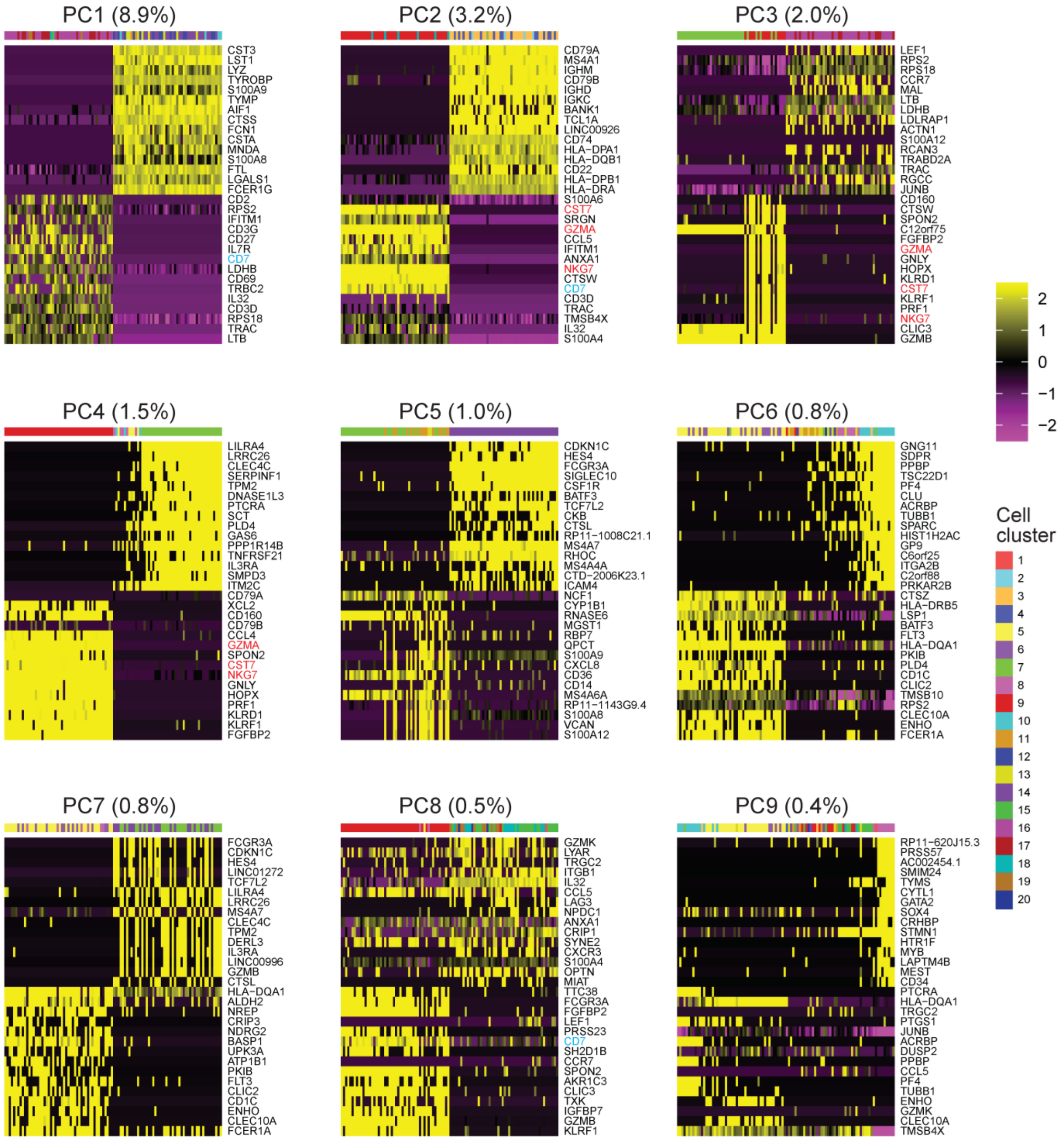

Figure S10. Genes can be highly correlated with many PCs from PCA. Heatmaps of the first 9 PCs colored by scaled normalized gene expressions. Genes are ranked by their loadings in increasing order. The top and bottom 15 genes for each PC are shown. Top annotation row indicates a total of 100 cells with the highest and lowest PC scores and are colored by Celda cell cluster labels as in figures 2 and 3. CST7, NKG7 and GZMA (highlighted in red) are present in the top genes in PCs 2, 3, and 4. CD7 (highlighted in cyan) is present in the top genes in PCs 1, 2, and 8.

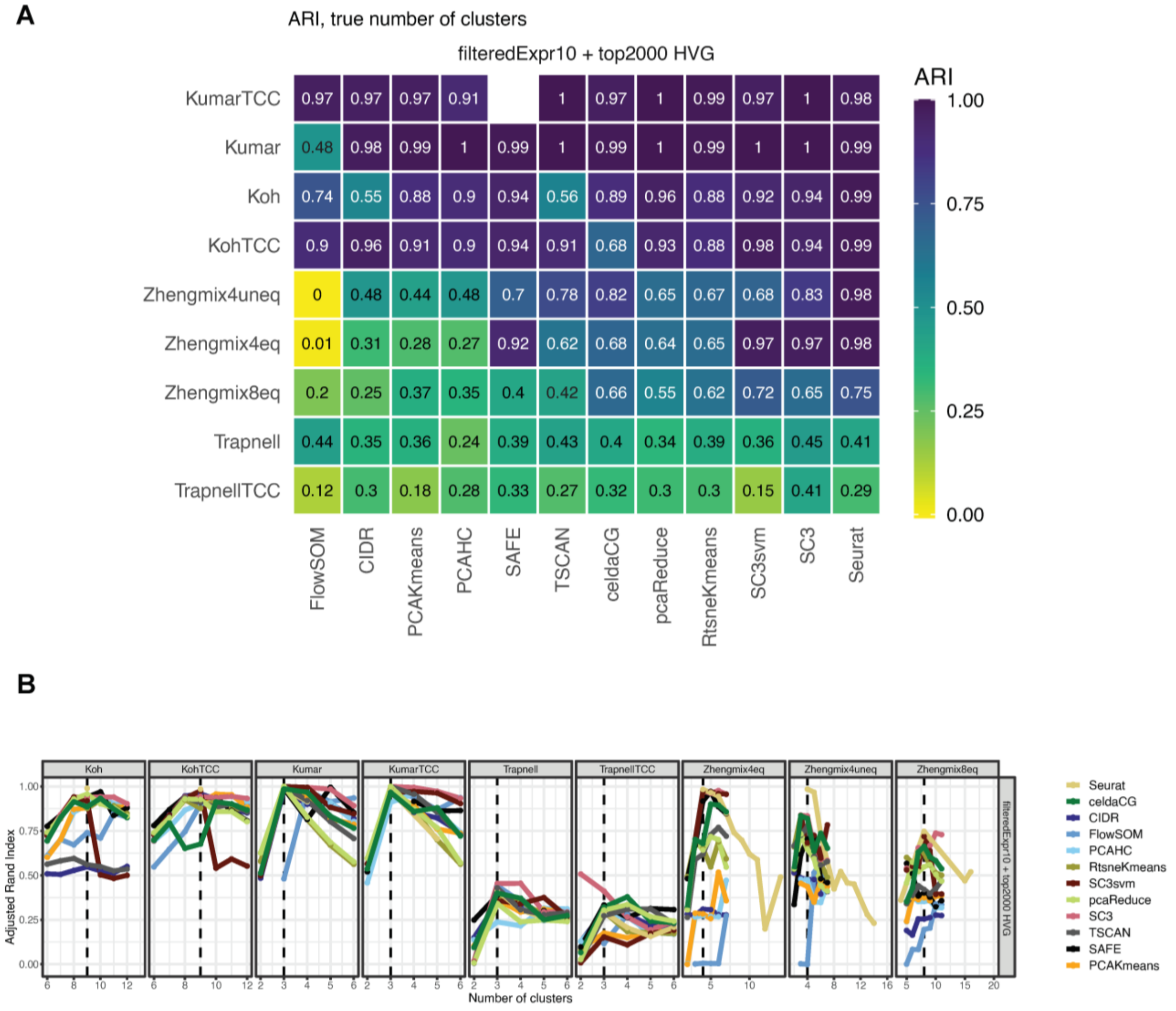

Figure S11. The cell clustering performance of Celda is comparable to other methods. a, Heatmap showing the ARIs of clustering results with respect to true partition, with the number of clusters fixed to the true number. Each of the 9 datasets were evaluated using Celda_CG and 11 other algorithms. Based on median ARI across all datasets, Celda ranked 6 out of 12 algorithms tested. However, Celda performed within 0.1 median ARI of the top algorithm in 6 of the 9 datasets suggesting the cell clustering accuracy is relatively close to other tools. Some methods failed to return a clustering with the correct number of clusters for certain data sets (indicated by white squares). **b**, ARIs of each algorithm's clustering results with respect to true partitions, under varying number of cell clusters. In the KohTCC and Zhengmix4eq datasets, performance of Celda increased with the addition of one extra cluster.

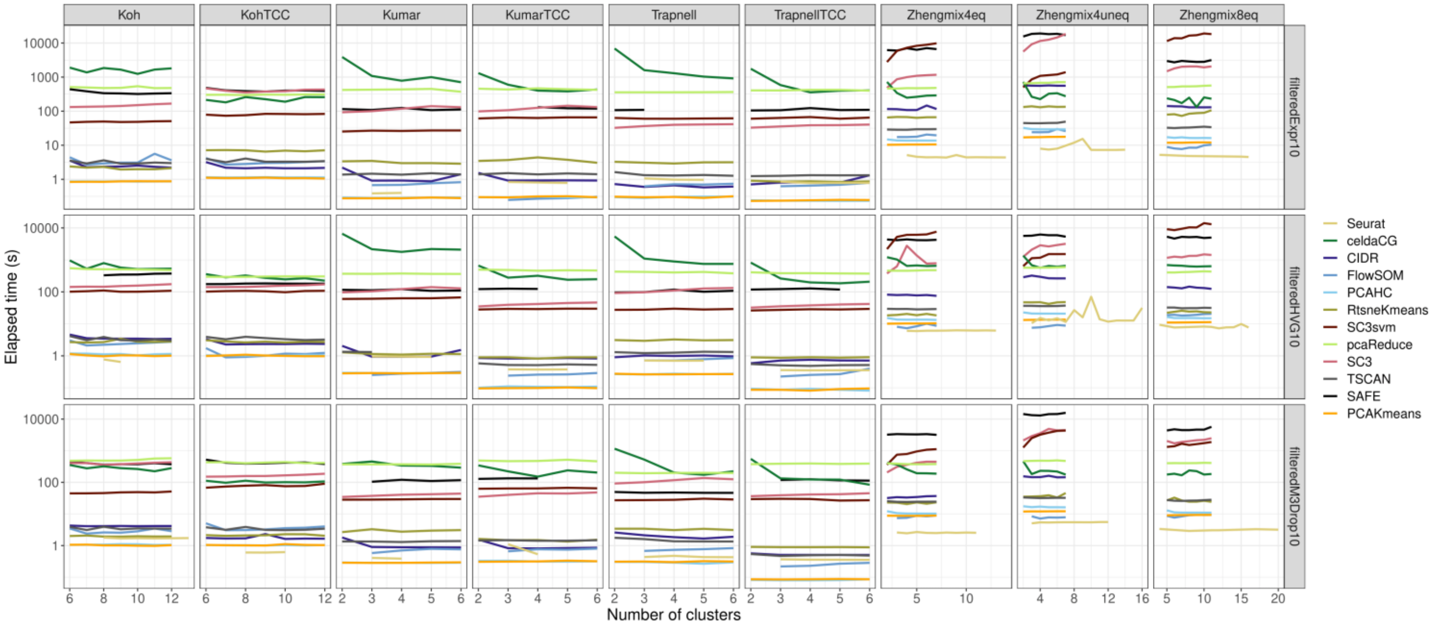

Figure S12. Run times for 12 methods on 9 datasets in DuoClustering2018 benchmark. Run times for tested scRNA-seq clustering algorithms with varying numbers of assigned cell clusters are shown.

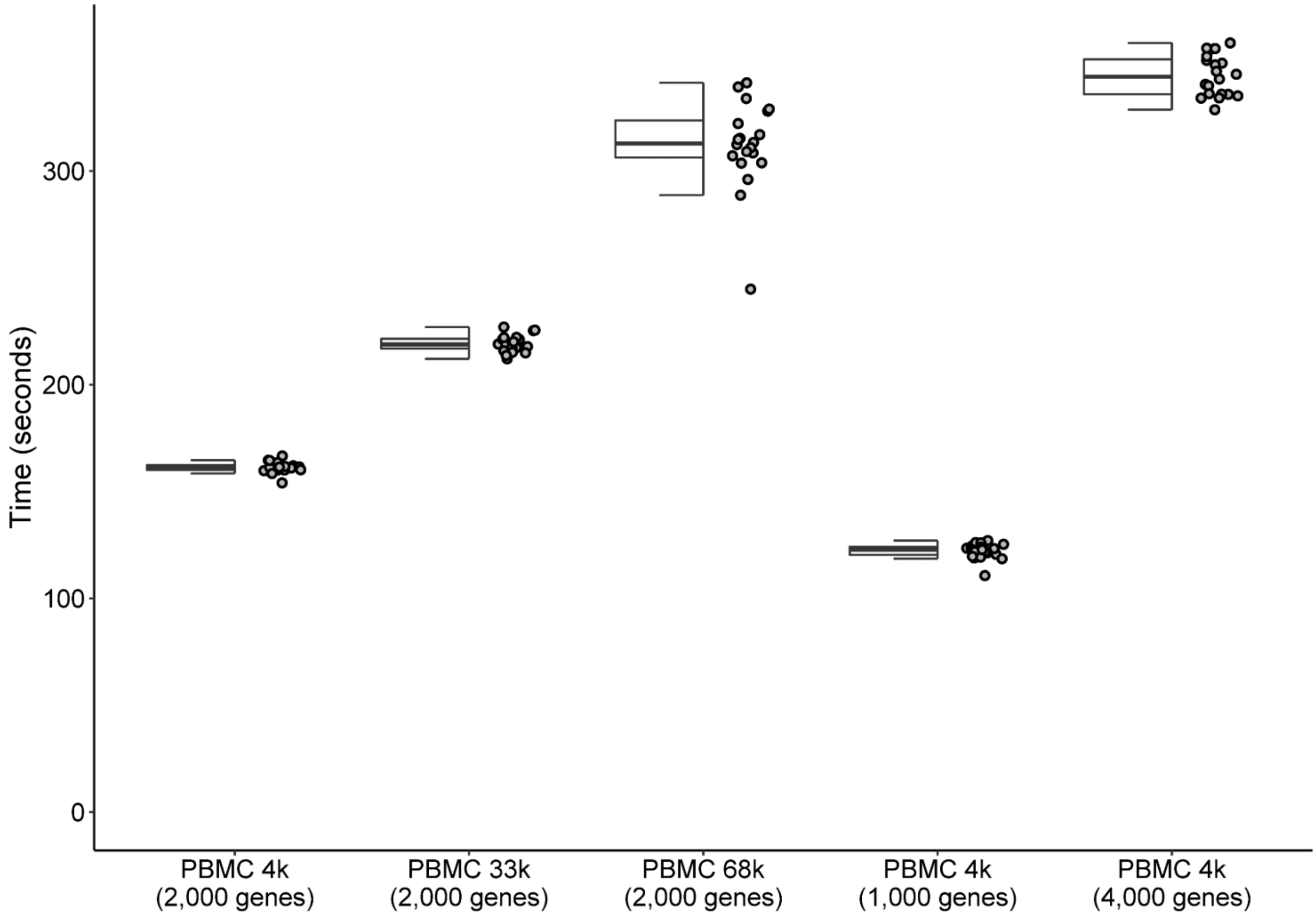

Figure S13. The speed of Celda_CG clustering scales proportionately with dataset size. Box and dot plots of the elapsed times of 20 replicates for PBMC 4k, 33k, and 68k datasets are shown. Median run times of Celda_CG were 161.3, 218.8, and 312.9 seconds for 4k, 33k, and 68k datasets respectively.
